# Provider REport of Sustainment Scale (PRESS): development and validation of a brief measure of inner context sustainment

**DOI:** 10.1186/s13012-021-01152-w

**Published:** 2021-08-30

**Authors:** Joanna C. Moullin, Marisa Sklar, Mark G. Ehrhart, Amy Green, Gregory A. Aarons

**Affiliations:** 1 Faculty of Health Sciences, Curtin Medical School, Curtin University, Kent Street, Bentley, Perth, Western Australia 6102 Australia; 2 Child and Adolescent Services Research Center, 3665 Kearny Villa Rd., Suite 200N, San Diego, CA 92123 USA; 3 Department of Psychiatry, University of California San Diego, 9500 Gilman Drive (0812), La Jolla, CA 92093-0812 USA; 4 UC San Diego ACTRI Dissemination and Implementation Science Center (UC San Diego ACTRI DISC), Altman Clinical and Translational Research Institute (ACTRI), 9500 Gilman Drive (0990), La Jolla, CA 92093-0990 USA; 5 Department of Psychology, University of Central Florida, PO Box 161390, Orlando, FL 32816-1390 USA; 6 The Trevor Project, PO Box 69232, West Hollywood, CA 90069 USA

**Keywords:** Sustainment, Sustainability, Maintenance, Implementation, Measurement, Scale, Psychometric properties, Knowledge translation

## Abstract

**Background:**

Implementation scientists and practitioners often rely on frontline providers for reporting on implementation outcomes. Furthermore, measures of sustainment are few, and available sustainment measures are mainly setting or evidenced-based practice (EBP) specific, require organizational and system-level knowledge to complete, and often lack psychometric rigor. The aim of this study was to develop a brief, pragmatic, and generalizable measure for completion by frontline service providers of the implementation outcome, sustainment.

**Methods:**

We utilized a Rasch measurement theory approach to scale the development and testing of psychometric parameters. Sustainment items were developed to be relevant for direct service providers to complete. In order to promote generalizability, data were collected and items were tested across four diverse psychosocial evidence-based practices (motivational interviewing [MI], SafeCare®, classroom pivotal response training [CPRT], and an individualized mental health intervention for children with autism spectrum disorder [AIM-HI]) and in four service settings (substance use disorder treatment, child welfare, education, and specialty mental health). Associations between the sustainment measure and sustainment leadership, sustainment climate, and attitudes towards the adoption and use of each of the EBPs were assessed to confirm construct validity.

**Results:**

Three items for the Provider REport of Sustainment Scale (PRESS) were assessed for measuring the core component of sustainment: continued use of the EBP*.* Internal consistency reliability was high. The scale indicated fit to the Rasch measurement model with no response dependency, ordered thresholds, no differential item functioning, and supported unidimensionality. Additionally, construct validity evidence was provided based on the correlations with related variables.

**Conclusion:**

The PRESS measure is a brief, three-item measure of sustainment that is both pragmatic and useable across different EBPs, provider types, and settings. The PRESS captures frontline staffs’ report of their clinic, team, or agency’s continued use of an EBP. Future testing of the PRESS for concurrent and predictive validity is recommended.

Contributions to the literature

- This study provides a brief and pragmatic measure of sustainment for completion by frontline providers representing the clinic, team, or agency level, which shows good reliability and validity.
- The Provider REport of Sustainment Scale (PRESS) measures the continued use of an evidence-based practice.
- Provider REport of Sustainment Scale (PRESS) is designed to be adapted and used across different service settings and different evidence-based practices.

## Background

Without sustainment, the public health benefits of the implementation of evidence-based practices (EBPs) will be limited and the accumulated costs from EBP development, evaluation, and implementation are futile. Unfortunately, successful implementation does not guarantee ongoing sustainment [1–3]. In fact, most implementation efforts fail to make it to the sustainment phase [3–6]. The influences and strategies to promote an EBP’s sustainment may be unique or distinct from influences and strategies that are effective in previous stages and thus require additional tools and research approaches [7, 8]. Despite acknowledgement of the importance of sustainment, there are multiple reasons why this final phase of the implementation process is less well studied, one of which being a lack of valid, easily accessible, pragmatic measures of sustainment that are appropriate for direct service providers [9–11].

Before moving forward, it is important to note the distinction between sustainment and sustainability. At its core, sustainment addresses the continued use of the practice that is the target of the implementation, whereas sustainability addresses whether the factors are in place to promote that ongoing use. Thus, sustainment can be considered “an outcome of a sustainability effort” [12].

The measurement of sustainment and sustainability has been an ongoing issue in the implementation science literature. Although there are a number of published measures of sustainment and sustainability, existing measures tend to address sustainability rather than sustainment and tend to focus on community public health programs (e.g., smoking cessation campaigns) rather than specific EBPs implemented at an organizational level [8]. In addition, a recent review of published measures of sustainment and sustainability found existing measures tend to be time-intensive, require stakeholders across multiple levels or stakeholders with knowledge across multiple levels to complete, and tend to be limited to assessing a particular EBP or only appropriate for a specific setting [9]. As an example, recently, a 35-item Sustainment Measurement System Scale (SMSS) [11] was published to assist Substance Abuse and Mental Health Services Administration (SAMHSA)-funded preventative programs. Although this measure was developed with a rigorous process and has a number of strengths, one challenge is that the questionnaire includes the perspective of government administrators and organizational executives who are aware of the “legislation, policies, public-sector fiscal resource availability, bid solicitations, reimbursement schemes, and how these factors are instantiated into service contracts.” [13]. Those same individuals may lack intimate knowledge of the day-to-day practice in the clinic. Thus, although such measures have their place and can be useful for a number of purposes, they do not address the providers’ assessment of what is happening at the ground level of implementation. Furthermore, they are often too lengthy and involved for many purposes, such as providing a quick and pragmatic assessment of the current state of sustainment. There appears to be no measure that is applicable across health care settings, that can be tailored for particular EBPs, and that is designed to be completed by frontline providers [9].

Our knowledge and understanding of sustainment would benefit from additional measures that are valid, reliable, suitable, and accessible indicators of whether an EBP continues to be delivered. Brief and pragmatic measures of the implementation process are needed to advance implementation theory, identify the mechanisms associated with the sustained use of EBPs, and measure implementation effectiveness [9, 14]. This study is consistent with conceptual models and implementation frameworks such as the Exploration, Preparation, Implementation, Sustainment (EPIS) framework that identify multiple phases or stages in the implementation process [15–18] and include focus explicitly on the sustainment phase [19, 20].

The aim of this study was to develop a very brief, pragmatic, valid, and reliable measure of an EBP’s sustainment from the perspective of service providers targeting the inner context organizational and provider levels. Specifically, we aimed to (1) generate sustainment items and assess items’ face and content validity; (2) conduct Rasch analyses to assess construct validity and item functioning; (3) examine potential demographic, contextual, and nested structure influences (e.g., EBP, position, race, and ethnicity) on item functioning; and (4) examine construct validity by assessing relationships between the sustainment measure and other constructs expected to be associated with sustainment, including sustainment climate, sustainment leadership, and attitudes towards the specific EBP.

## Methods

Scale development proceeded following the approach described by DeVellis which consists of (i) defining the construct to be measured, (ii) generating items and the response format by an expert panel, (iii) administering items to a sample, and (iv) evaluating items and optimizing the scale length [21]. We evaluated items using Rasch measurement theory. We also followed Glasgow and Riley’s [14] call for the development of pragmatic measures. Table 1 provides the Glasgow and Riley [14] recommendations for pragmatic measures and how each was addressed in the study.

**Table 1:** Required and recommended criteria for pragmatic measures

Glasgow and Riley criteria	Provider REport of Sustainment Scale (PRESS)
Important to stakeholders	Items and constructs were reviewed by practitioners, supervisors, and other researchers before data collection
Burden is low for both respondents and staff	Measure is freely available and takes less than 5 min to complete
Actionable	Easy to score and interpret
Sensitive to change	Items are phrased to be sensitive to change and valid across the spectrum of sustainment
Broadly applicable	Can be used across different settings and EBPs
Use for benchmark, has norms to interpret, or addresses public health goals	Informs sustainment of a practice in specific settings so that norms can be developed and addresses public health
Unlikely to cause harm	No sensitive information collected
Psychometrically strong	Rasch Measurement Theory and Classical Test Theory support reliability and validity
Related to theory or model	Covers constructs of EPIS model and existing constructs and definitions of sustainment

### Definitions

We utilized the core component of the definition of sustainment, “the input(s) (e.g., intervention, program, implementation strategy) continue to be delivered” [9], and focused on actions or processes that would be observable and could be reported on by frontline providers.

Evidence-based practice is defined as “the integration of the best available research with clinical expertise in the context of patient characteristics, culture, and preferences.” [22]. This study addresses the sustainment of the specific treatment and interventions that align with that definition (e.g., motivational interviewing). Our focus on EBPs is driven by the shortage of applicable sustainment measures that can be applied in implementation studies of specific interventions proven to improve outcomes and within different service delivery contexts.

### Provider REport of Sustainment Scale (PRESS) item generation and response format

A review of the literature was conducted to create a catalogue of existing measures used in empirical research on sustainment [9]. Constructs represented across all included measures were reviewed and synthesized to facilitate the selection and creation of items assessing continued use. Initially, 5 items were generated by a subset of the authors that targeted EBP usage and provider competence. To strengthen face validity, content validity, clarity, and relevance of the scale, items were reviewed by another subset of authors (*n* = 3) and other stakeholders (*n* = 3) of clinical and organizational psychologists with expertise in implementation science as well as by stakeholders in service settings implementing EBPs, including clinicians. Each reviewer was asked to provide general feedback on the individual items as well as whether additional items should be included. This was a multistep, iterative process until consensus was formed on the items.

To align with other widely used implementation measures, a 5-point Likert-type scale was used across all items such that responses ranged from 0 = not at all to 4 = to a very great extent. This response format has been used in multiple implementation measures, including those included to assess construct validity: sustainment climate scale [23], sustainment leadership scale [24], and the Evidence-Based Practice Attitude Scale (EBPAS) [25].

Although they varied slightly across the four samples used in this paper, the basic instructions were as follows: “The following questions ask about [EBP] in your [team/agency/school]. Please indicate the extent to which you agree with the following items where 0-Not at all, 1- to a slight extent, 2-to a moderate extent, 3-to a great extent, 4-to a very great extent.”

### Participants

Items were administered to participants of four existing US National Institutes of Health (NIH) funded studies: (1) a cluster randomized control trial of the Leadership and Organizational Change for Implementation (LOCI) strategy in substance use disorder treatment (SUDT) agencies implementing motivational interviewing [26], (2) a mixed-methods study of EBP sustainment in a state and county child welfare service systems implementing SafeCare® [13], and (3 and 4) the Translating Evidence-Based Interventions for Autism Spectrum Disorder: multilevel implementation strategy (TEAMS) study [27]. The TEAMS study looked at the effectiveness of an implementation strategy across two studies that take place in public mental health programs implementing an individualized mental health intervention for autism spectrum disorder, or schools implementing classroom pivotal response teaching (CPRT). In each study, participants were eligible to complete the surveys if they were direct providers of the EBP being implemented or supervisors of those providers.

### Survey procedure

Via the Qualtrics web-based platform, respondents completed surveys between 1 and 5 years following initial EBP implementation. Surveys remained accessible for approximately 1 month such that providers could respond at a time and place of their convenience. Back-up data collection procedures were in place including e-mail follow-up reminders. Consent was obtained from service providers to participate in ongoing surveys of the studies. All studies were approved by the appropriate Institutional Review Boards.

### Measures

#### Demographics

Demographic information was collected as part of all of the studies including age, gender, race/ethnicity, and position.

#### Sustainment Climate Scale (SCS)

The implementation/sustainment climate measure [23] includes 18 items that assess the degree to which there is a strategic organizational climate supportive of EBP implementation/sustainment. The overall scale in the development study had a Cronbach’s alpha of 0.912 and contains the following six subscales: focus, educational support, recognition, rewards, selection for EBP, and selection for openness. This scale was included to assess construct validity because an organizational environment supportive of sustainment was expected to be positively associated with sustainment.

#### Sustainment Leadership Scale (SLS)

The implementation/sustainment leadership scale [24] includes 17 items that assess strategic leadership for EBP implementation/sustainment. The measure has an overall Cronbach’s alpha of 0.97 and includes the following four subscales: proactive, knowledgeable, supportive, and perseverant. This scale was included to assess construct validity because leadership supportive of sustainment was expected to be positively associated with sustainment.

#### Evidence-Based Practice Attitude Scale (EBPAS)

The Evidence-Based Practice Attitude Scale [25] includes 15 items that assess provider attitudes toward adoption of EBP generally. The measure has an overall Cronbach’s alpha of .76 and subscale alphas range from .66 to .91. The measure has been adapted to assess attitudes regarding the adoption of each of the specific EBPs targeted in the parent studies. The measure consists of the following four subscales: requirements, appeal, openness, and divergence [28]. This scale was included to assess construct validity because more positive attitudes towards the EBP were expected to be positively associated with sustainment.

### Data analyses

Data were downloaded into IBM SPSS V25 for data management and preliminary analyses. Initial data were screened for missing data and out-of-range values. Data were cleaned and variable distributions evaluated at both the univariate and multivariate levels.

For Rasch analysis, a sample size of 150 participants is sufficient to calibrate items to within ± 0.5 logits (*α* of 0.01 and *β* of 0.2) [29, 30] and with the same power to test for differential item functioning (DIF), where a difference of 0.5 standard deviations within the residuals can be detected for any two groups.

Internal consistency reliability coefficients [21] were calculated to assess reliability using Cronbach’s alpha and McDonald’s omega [31–33]. The Person Separation Index of the Rasch measurement model was also used, which is equivalent to Cronbach’s alpha using the logit value as opposed to the raw score and interpreted in the same way.

Data were fitted to the Rasch measurement model for polytomous response scales [34, 35] using RUMM2030 software [36] and following procedures consistent with key Rasch papers [37–39]. These procedures include assessing (a) fit to the model via fit statistics (item fit residual standard deviations less than 2.5, person fit residual standard deviations less than 1.5, and a non-significant total chi-square item-trait interaction statistic); (b) ordered item thresholds to determine the appropriateness of items’ response options; (c) strategies to improve fit (e.g., item deletion), and subsequent fit statistics; (d) assumption of local independence of items (response dependency with correlations below 0.3 and unidimensionality of scale); (e) generalizability/invariance via examination of differential item functioning (DIF); (f) targeting of the scale; and (g) person separation reliability (as described in the previous paragraph) [39]. The responses to the items were checked to ensure they fit with the metric estimates by analyzing category response/probability curves and category response frequencies. Appropriate steps were taken to order any disordered threshold found, such as collapsing categories, or removing persons or items with poor fit. Bonferroni corrections were applied to adjust the alpha value to take into account multiple testing, by dividing the alpha value of 0.05 by the number of items in the scale (3). To check the assumption that there is no pattern of item residuals after extraction of the latent variable, a residual correlation matrix was assessed. Any items indicating response dependency were combined into a subtest. Secondly, unidimensionality of the scale was tested through conducting a principal component analysis of item residuals, performing a test of two subsets of items based on residual patterns, and performing an independent samples *t*-test. No pattern of residuals supported the assumption of unidimensionality. Lack of differential item functioning (DIF) was used to establish measurement invariance across EBP/settings and demographic variables (e.g., gender, race/ethnicity). The mean “location” score of persons was compared to the item’s location to assess how well-targeted the scale was for the sample, with a mean location around zero indicating a well-targeted scale and a positive mean value indicating the sample as a whole was located at a higher level of sustainment than the average of the scale, while a negative value suggesting a lower level.

Finally, correlations between the PRESS sustainment measure aggregated scores and the SCS, SLS, and EBPAS were examined to confirm construct validity. Specifically, the PRESS, SCS, and SLS scale scores were considered as representing unit-level constructs and aggregated to the work group level (school district, clinical group, team) [24, 25, 40, 41, 46]. Intraclass correlation coefficients (ICCs) for work group aggregated scale scores were .50, .22, and .32 for the PRESS, SCS, and SLS, respectively. The EBPAS was similarly aggregated to the work group level. ICCs for EBPAS scales were .03, .12, .09, and .05 for the Divergence, Openness, Appeal, and Requirements subscales, respectively. All scale scores were mean deviated, and correlations between centered scores were examined. We expected a moderately sized correlation between the PRESS and SCS and SLS and small to moderately sized correlations between the PRESS and EBPAS.

## Results

After four iterations with the stakeholder review panel, three items were generated representing continued use of an EBP, analyzed as the PRESS, with two items being removed as they related to fidelity. The resulting PRESS items are shown in Table 2.

**Table 2:** Provider REport of Sustainment Scale (PRESS)

*The following questions ask about [EBP] in your [setting].* *Please indicate the extent to which you agree with the following items*	
1. Staff use [EBP] as much as possible when appropriate	
2. Staff continue to use [EBP] throughout changing circumstances	
3. [EBP] is a routine part of our practice	

Anchors 0 = not at all, 1 = to a slight extent, 2 = to a moderate extent, 3 = to a great extent, and 4 = to a very great extent

We received 527 responses to the scale. The distribution of the surveys varied across studies, with declinations not recorded for SafeCare® as it was collected in the field and as such the overall response rate is unknown. Participants were supervisors (*n* = 59) or service providers (*n* = 468 clinicians, case managers, and teachers). Participants provided motivational interviewing in substance use disorder treatment (SUDT) (*n* = 121), SafeCare® in child welfare (*n* = 211), classroom pivotal response teaching (CPRT) in education (*n* = 109), or an individualized mental health intervention for autism spectrum disorder (AIM-HI) in mental health (*n* = 86) services in multiple counties across California and Oklahoma.

The sample (see Table 3) was 87.6% female, 35.0% Hispanic or Latino origin, 62.2% White, 9.6% Black or African American, 4.8% Asian, 3.5% American Indian/Alaskan Native, 2.1% Native Hawaiian/other Pacific Islander, 6.4% were more than one race, and 11.4% as others. In total, 24.1% were 20–29 years of age, 32.5% were 30–39 years, 20.2% were 40–49 years, and 23.2% were 50 years or older.

**Table 3:** Participant characteristics

Characteristic	Number (%)
Gender	
Male	65 (12%)
Female	461 (88%)
Missing/not reported	1
Age	
20–29 years	124 (24.1%)
30–39 years	167 (32.5%)
40–49 years	104 (20.2%)
50 plus	119 (23.2%)
Missing/not reported	13
Race	
White	323 (62.2%)
Black or African American	50 (9.6%)
Asian	25 (4.8%)
America Indian/Alaskan Native	18 (3.5%)
Native Hawaiian/other Pacific Islander	11 (2.1%)
More than one race	33 (6.4%)
Others	59 (11.4%)
Missing/not reported	8
Ethnicity	
Hispanic or Latino	184 (35.0%)
Missing/not reported	1
Position	
Supervisor	59 (11.2%)
Provider	468 (88.8%)

Internal consistency was high (Cronbach’s alpha = 0.947; McDonald’s omega = .948) and 85.8% of the variance in the items was explained by the PRESS scale. The mean inter-item correlation was 0.858. Internal consistency as expressed in Rasch was very good with a PSI of 0.826 with extremes included (people that endorsed all items with a zero or all items with a four) or 0.845 with no extremes [21].

Data were checked for suitability for Rasch analyses. There were no missing values for any item and the minimum and maximum responses were endorsed for all items. The scale indicated fit to the Rasch measurement model with the total chi-square item-trait interaction statistic (19.441) being non-significant (*p* = 0.078) and fit residual standard deviations for items (0.766) and persons (1.458) less than then recommended 2.5 and 1.5, respectively [42] (see Table 4). There appeared to be minimal response dependency, with correlations between all items below 0.3. Thresholds were ordered and no uniform or non-uniform DIF were found across EBP, race, age, gender, or position. Independent samples *t*-tests comparing the person trait estimated on the two most divergent items (item 1 and item 3) showed significant difference (*p* < 0.05) in scores for only 13 of the 527 tests (2.47%), providing support of unidimensionality of the scale [42].

**Table 4:** Summary of results of Rasch analysis of sustainment within the inner context measure

Analysis	Overall model fit	Item fit residual mean (SD)	Person fit residual mean (SD)	PSI [a]	% Sig. ***t*** tests
1. Items 1, 2, 3	*χ*^2^ = 19.441, df = 12, *p* = 0.078	0.766	1.458	0.826	2.47%

*SD* standard deviation, *χ2* chi-square, *df* degrees of freedom, *p* probability, *PSI* Person Separation Index

[a] PSI with extremes included (0.8445 without extremes)

**Fig. 1:**
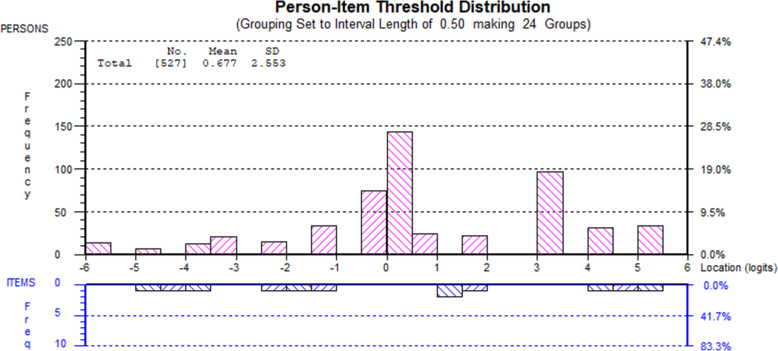
Person-item threshold distribution (extremes included) [*n* = 527]

The data showed variability in sustainment with the mean person logit scores varying across the studies and EBP being implemented. The mean person logit score was 0.677, SD = 2.553 (see Fig. 1). Participants implementing SafeCare® had a mean logit score of 1.632, SD = 2.05; MI a mean logit score of 0.912, SD =2.26; CPRT a mean logit score of −0.302, SD = 2.75; and AIM-HI a mean logit score of −0.72, SD 2.74. One-way analysis of variance (ANOVA) of total scores indicated significant differences in mean/total sustainment by EBP (*F*(3, 523) = 149.23, *p* < .001). Special follow-up contrasts were examined to better characterize between EBP differences in sustainment. Significantly greater sustainment was found with SafeCare® ($\overline{x}$ = 3.64) than MI ($\overline{x}$ = 2.58), CPRT ($\overline{x}$ = 2.00), and AIM-HI ($\overline{x}$ = 1.89) combined (*t*(526) = 20.42, *p* < .001). Significantly greater sustainment of MI than CPRT and AIM-HI combined was found (*t*(526) = 26.78, *p* < .001). There were no significant differences in sustainment between CPRT and AIM-HI (*t*(526) = 0.90, *p* = .369). Age also had a significant effect (*F* (3, 405) = 3.72, *p* = 0.012). Overall, participants aged 20 to 29 years had a mean logit score of 0.521, SD = 2.54; those aged 30 to 39 years a mean logit score of 0.338, SD = 2.7; those aged 40 to 49 0.665, SD = 2.35; and those aged 50 and over a mean of 1.581, SD = 2.58. Mean sustainment did not significantly differ between supervisors (mean logit score 1.126, SD = 2.02) and providers (mean logit score 0.621, SD = 2.61). The total score statistics held when extreme persons (those who endorsed all items either 0 or 4) were removed (*n* = 157). Response options for items ranged across the sample’s person location distribution (see Fig. 1).

Significant, positive correlations of varying magnitudes were found between the PRESS and all scales with the exception of the EBPAS requirements subscale (*r*(66) = .166, *p* = .182). Correlations between the PRESS and the SCS scales varied; correlations were the smallest for reward (*r* (98) = .342, *p* = .001) and greatest for overall sustainment climate (*r* (98) = .636, *p* < .001). Correlations between the PRESS and the SLS were generally stronger than those with the SCS. Correlations between the PRESS and the SLS scale were the smallest for knowledge (*r* (66) = .606, *p* < .001) and greatest for proactive (*r* (66) = .779, *p* < .001). Overall, correlations between the PRESS and the EBPAS were comparatively weaker than those with the SCS and SLS. Of the significant correlations between the PRESS and EBPAS scales, magnitudes were smallest with divergence (*r* (66) = −.274, *p* = .026) and greatest for appeal (*r* (66) = .464, *p* < .001). See Table 5 for correlations.

**Table 5:** Correlation between Provider REport of Sustainment Scale (PRESS) and Sustainment Leadership Scale (SLS), Sustainment Climate Scale (SCS), and the Evidence-Based Practice Attitudes Scale (EBPAS) scores

	Mean	Std. Dev	1	2	3	4	5	6	7	8	9	10	11	12	13	14	15	16	17
1. PRESS	2.77	1.10																	
2. SLS Proactive	3.01	1.06	.78**																
3. SLS Knowledge	3.14	1.01	.616**	.85**															
4. SLS Support	3.26	0.91	.77**	.82**	.84**														
5. SLS Perseverant	3.24	0.93	.78**	.86**	.84**	.92**													
6. SLS Total	3.16	0.90	.78**	.94**	.94**	.94**	.96**												
7. SCS Focus	3.16	0.87	.61**	.84**	.64**	.74**	.78**	.80**											
8. SCS Edu Supt	2.66	1.14	.437**	.73**	.59**	.62**	.67**	.70**	.74**										
9. SCS Recognition	2.18	1.08	.37**	.54**	.38**	.40**	.44**	.47**	.54**	.54**									
10. SCS Rewards	1.04	1.26	.34**	.11	.01	.03	.07	.06	.14	.27**	.58**								
11. SCS Select for EBP	2.44	1.15	.49**	.70**	.59**	.58**	.70**	.68**	.53**	.49**	.54**	.31**							
12. SCS Select for Open	2.90	0.99	.44**	.68**	.57**	.61**	.67**	.67**	.48**	.46**	.47**	.33**	.82**						
13. SCS Total	2.44	0.83	.64**	.80**	.62**	.67**	.75**	.76**	.77**	.77**	.75**	.53**	.85**	.81**					
14. EBPAS Require	2.9	0.95	.17	.01	.09	.15	.10	.08	.03	.15	.12	.09	−.23	−.20	−.04				
15. EBPAS Appeal	3.11	0.74	.46**	.30*	.33**	.41**	.40**	.38**	.38**	.29*	.42**	.26*	.12	.14	.30*	.62**			
16. EBPAS Openness	3.22	0.67	.33**	.30*	.29*	.32**	.33**	.33**	.37**	.17	.34**	.19	.07	.14	.23	.29*	.73**		
17. EBPAS Divergence	1.03	0.99	−.27*	−.08	−.05	−.16	−.08	−.10	−.18	.13	.15	.41**	.19	.12	.14	−.19	−.32**	−.31*	
18. EBPAS Total	3.05	0.56	.40**	.21	.24	.34**	.28*	.28*	.30*	.14	.21	.01	−.10	−.04	.08	.72**	.87**	.73**	−.65**

*PRESS* Provider REport of Sustainment Scale, *SLS* Sustainment Leadership Scale, *SCS* Sustainment Climate Scale, *EBPAS* Evidence-Based Practice Attitudes Scale

**p < .01, *p < .05

Grand means and standard deviations are presented in the first two columns

## Discussion

This study developed and tested a three-item sustainment outcome measure on the use of an EBP suitable for frontline service providers delivering EBPs in the inner context of clinics, teams, and organizations. Internal consistency reliability estimates were high. The scale indicated fit to the Rasch measurement model with no response dependency, ordered thresholds, no differential item functioning, and supported unidimensionality. The PRESS was designed to be usable across different health care and organizational settings, as well as to be tailored for specific EBPs. The PRESS was tested across four diverse EBPs (motivational interviewing, SafeCare®, classroom pivotal response training, and an individualized mental health intervention for children with autism spectrum disorder) and four settings (substance use disorder treatment, specialty mental health, child welfare, and education). By collecting data across these studies, we expanded the variability in agencies, programs, disciplines, settings, and EBPs, thus increasing the generalizability, utility, and its potential to impact implementation science and improving public health across sectors.

All participants had been involved in the implementation of their EBP for at least 12 months; however, the data sets represent different time frames for when the projects started and then moved to the sustainment phase. The EBPs ranged from what could be described as not yet sustained to sustained. In particular, the participants in the SafeCare® study had been delivering the practice for 5 years or more and the practice was well integrated into their organizations and routine practice. This was apparent in the results of the scale for this sector. However, it is important to note that the scale also performed well for those where the practice was earlier in the implementation process.

The PRESS complements other measures. As an example, the Sustainment Measurement System Scale (SMSS) is an outcome measure for evidence-based practice broadly (rather than sustainment of a particular EBP) and includes outer contextual knowledge about funding [11]. The PRESS measure on the other hand is specific for the outcome of continued use of a specific EBP and focuses on the provider observation of EBP use, making it pragmatic, generalizable, and suitable for use by practitioners, purveyors, and researchers alike.

The ultimate goal of improving any implementation factor is to increase the implementation and later sustainment of the EBP. With a valid and reliable measure, sustainment can be treated as a dependent variable or as a mediator or moderator of other implementation (e.g., reach/penetration, fidelity) or clinical outcomes (e.g., patient symptoms/behaviors) across studies, facilitating experimental studies of implementation strategies, delineating their mechanisms of action, and potentially contributing to systematic reviews and meta-analyses across studies.

### Future recommendations

Because the sustainment measure was developed to be generalizable across settings, it does not measure the full spectrum of EBP continuation. Furthermore, the measure was used in conjunction with research-supported implementation/sustainment efforts. It is possible PRESS may function differently in more “real-world” implementation/sustainment efforts. Components of the sustainment variable that are EBP specific, notably sustained fidelity and EBP outcomes (e.g., patient or provider benefits) require tailored assessment. In addition, measures to be completed by higher management or executives relating to outer context sustainment (e.g., funding stability) are suited to a separate measure to ensure respondents are able to answer accurately (e.g., the SMSS [11]). Our recommendation is that PRESS is used in conjunction with other assessments of these components. We also recommend that validation in additional contexts and predictive validity to clinical or service outcomes be evaluated in future research. In terms of criterion validity, the PRESS is based on self-report, Likert-type scale, which may not be as objective a measurement as observation; however, it is the benefit of being pragmatic and feasible.

The scale was developed for providers involved in team/agency-level EBP implementation projects. As such, the referent “staff” was used in the items, but equally the referent “our team” may be applicable and consistent with the goal of the scale as developed. The ICC statistics supported aggregation of the PRESS scale to the work group unit level (school district, clinical group, team). Through shared experiences and communication throughout the work group, individual staff likely have similar ideas about the overall sustainment levels across the work group as a whole. Thus, the aggregate scores provide a meaningful indicator of the overall sustainment levels in the work group.

There are occasions when a focus at lower levels may be of primary interest, and there is the possibility for the PRESS items to be adapted for use at an individual level (e.g., physician or patient). For example, items may be adapted for self-ratings of sustainment, such as “I use the [EBP/intervention] as much as possible; (2) I continue to use [EBP/intervention] throughout changing circumstances; (3) The [EBP/intervention] is a routine part of my life.” Such a scale could be used to measure the sustainment of individual behavioral change interventions such as mental health, diet, and lifestyle. Similarly, a self-rated version could be suitable for implementation projects in settings where team-level aggregation is not appropriate (e.g., primary care physicians in private practice). As always, such adaptations would require additional psychometric testing.

## Conclusion

The Provider REport of Sustainment Scale (PRESS) responds to the need for a brief, pragmatic, reliable, and valid measure of EBP sustainment [9]. The measure will provide a better understanding of the sustained use of EBPs in organizational settings and subsequently enhance the persistence of intended improvements in patient/client outcomes.

## Data Availability

Available upon request of the principal of each of the studies that provided data for the measure development.
